# Gene Expression Analysis before and after Treatment with Adalimumab in Patients with Ankylosing Spondylitis Identifies Molecular Pathways Associated with Response to Therapy

**DOI:** 10.3390/genes8040127

**Published:** 2017-04-24

**Authors:** Marzia Dolcino, Elisa Tinazzi, Andrea Pelosi, Giuseppe Patuzzo, Francesca Moretta, Claudio Lunardi, Antonio Puccetti

**Affiliations:** 1 Immunology Area, Pediatric Hospital Bambino Gesù, 00146 Rome, Italy; andrea.pelosi@opbg.net (A.P.); antonio.puccetti@opbg.net (A.P.); 2 Department of Medicine, University of Verona, 37134 Verona, Italy; elisa.tinazzi@univr.it (E.T.); jeps.pz@gmail.com (G.P.); francescamoretta82@gmail.com (F.M.); claudio.lunardi@univr.it (C.L.)

**Keywords:** Ankylosing spondylitis, Adalimumab (ADA), gene-array, protein-protein interaction (PPI) network, gene module

## Abstract

The etiology of Ankylosing spondylitis (AS) is still unknown and the identification of the involved molecular pathogenetic pathways is a current challenge in the study of the disease. Adalimumab (ADA), an anti-tumor necrosis factor (TNF)-alpha agent, is used in the treatment of AS. We aimed at identifying pathogenetic pathways modified by ADA in patients with a good response to the treatment. Gene expression analysis of Peripheral Blood Cells (PBC) from six responders and four not responder patients was performed before and after treatment. Differentially expressed genes (DEGs) were submitted to functional enrichment analysis and network analysis, followed by modules selection. Most of the DEGs were involved in signaling pathways and in immune response. We identified three modules that were mostly impacted by ADA therapy and included genes involved in mitogen activated protein (MAP) kinase, wingless related integration site (Wnt), fibroblast growth factor (FGF) receptor, and Toll-like receptor (TCR) signaling. A separate analysis showed that a higher percentage of DEGs was modified by ADA in responders (44%) compared to non-responders (12%). Moreover, only in the responder group, TNF, Wnt, TLRs and type I interferon signaling were corrected by the treatment. We hypothesize that these pathways are strongly associated to AS pathogenesis and that they might be considered as possible targets of new drugs in the treatment of AS.

## 1. Introduction

Ankylosing spondylitis (AS) is a progressive and debilitating seronegative spondiloarthritis, involving primarily the sacro-iliac joints and the spine and frequently the peripheral joints. Extra-articular tissues involvement, such as skin, tendons or eyes is often present [1]. AS has a strong genetic background being associated with the major histocompatibility complex (MHC) class I allele HLA-B27 in more than 90% of the patients [2].

The disease prevalence ranges the 0.02%–0.35% depending on the race, with a higher prevalence in males than in females [1].

According to the Assessment of SpondyloArthritis International Society/European League Against Rheumatism (ASAS/EULAR) and American College of Rheumatology/ Spondylitis Association of America/ Spondyloarthritis Research and Treatment Network (ACR/SAA/SPARTAN), there is a recommendation for patients with a predominantly active axial manifestation of AS that they should be treated for at least three months with nonsteroidal anti-inflammatory drugs (NSAIDs) followed by a tumor necrosis factor (TNF)-alpha blocker in case of non response or of NSAIDs-related side effects, while patients with both axial and peripheral involvement need treatment with TNF-blockers and eventually with disease-modifying antirheumatic drugs (DMARDS) in case of intolerance or controindication to anti-TNF-alpha treatment [3,4].

Treatment with the TNF blockers, infliximab, etanercept, adalimumab, golimumab and certolizumab, leads to a good improvement in clinical symptoms, in C-reactive protein (CRP) levels and in magnetic resonance imaging (MRI)-detectable inflammation of the sacroiliac joints [5].

In particular, Adalimumab (ADA) is a humanized monoclonal antibody against TNF-alpha, able to block both monomeric and trimeric soluble forms of TNF-alpha and cell-surface TNF-alpha, inducing cell apoptosis.

In this work, we have analyzed the transcriptome of Peripheral Blood Cells (PBCs) from patients with AS in order to identify possible mechanisms involved in the pathogenesis of the disease.

Previous studies on gene expression analyses in PBCs have identified gene signatures specifically associated to several diseases including Rheumatoid Arthritis (RA) and Systemic Lupus Erythematosus (SLE) [6,7]. Moreover, in a previous work on microarray analysis in Psoriatic Arthritis (PsA), we analyzed Differentially expressed genes (DEGs) in PBCs and in cells derived from synovial biopsies, and found that gene expression profiles of the cells obtained from the inflamed synovium are similar to those of PBCs, indicating that PBCs can be a good substitute of the target tissues to analyze disease-associated gene expression [8].

High-throughput methodologies, including microarray, have increased our understanding of many diseases. However, while several studies on gene array analyses revealed transcriptional profiles of many single genes differentially expressed in AS compared to healthy subjects, such studies have not analyzed gene product interactions and molecular pathways possibly related to the disease onset. A careful evaluation of such networks can provide a deeper insight in the understanding of disease pathogenesis. In this study, we implemented the gene-array study with networks and modules analyses to uncover possible molecular pathways associated to AS and to a good response to ADA therapy.

This is indeed a novel approach since other studies used a conventional gene expression analysis to study, at gene level, the response to TNF inhibitors in rheumatoid arthritis [9,10] and to our knowledge, there are no such studies of ankylosing spondylitis.

In AS we found deregulated genes that are involved in important pathways; moreover, such genes are differently modulated in patients that show a good response to treatment compared to non-responders. Interestingly, in patients with a good response to treatment, we also observed that ADA normalized the altered expression of genes involved in AS-associated pathways.

## 2. Materials and Methods

### 2.1. Patients

We studied a cohort of ten patients (seven males and three females, mean age: 51.8 years) affected by Ankylosing spondylitis (AS), attending the Unit of Autoimmune Diseases, at the University Hospital of Verona, Italy. A written informed consent was obtained from all the partecipants to the study. The study was approved by the local Ethical Committee and all clinical investigations have been conducted according to the principles expressed in the Helsinki declaration.

All patients fulfilled the modified New York criteria for the diagnosis of AS which included radiological and clinical criteria represented by (1) radiological evidence of sacroileitis; (2) low back pain and stiffness standing for at least three months, improved by exercise and not relieved by rest; (3) decrease of lumbar spine motility in both sagittal and frontal planes; and (4) limitation of chest expansion [11,12].

The patients underwent clinical examination and laboratory evaluation, which included inflammatory markers, such as erytrocyte sedimentation rate (ESR) and C-reactive protein (CRP), and genetic screening for the association with the allele HLA-B27. All patients underwent conventional radiography, magnetic resonance imaging (MRI) for the axial and peripheric involvement of the arthropathy. All patients were treated with the same anti-TNF agent Adalimumab, and samples for the gene array analysis were collected before and seven days after the anti-TNF injection. Adalimumab was administrated every two weeks without any side effects. The clinical features of the patients are reported in Supplementary Materials Table S1.

The evaluation of responder and non-responder patients to the treatment was carried out according to the Bath Ankylosing Spondylitis Disease Activity Index (BASDAI score), defining remission when the score is less than 3.8 [13].

Ten healthy age and sex matched healthy subjects were used as controls.

### 2.2. Gene Array

Blood samples were collected using PAXgene Blood RNA tubes (PreAnalytiX, Hombrechtikon, Switzerland) and total RNA was extracted following the manufacturer instructions. cRNA preparation, samples hybridization and scanning were performed following the Affymetrix (Affymetrix, Santa Clara, CA, USA) provided protocols, by Cogentech Affymetrix microarray unit (Campus IFOM IEO, Milan, Italy). Human Genome U133A 2.0 (HG-U133A 2.0) Gene Chip (Affymetrix) were used. The HG-U133A 2.0 Gene Chip covers more than 22,000 probe sets corresponding up to 14,500 well-characterized human genes. The microarray analysis was carried out using the Gene Spring software, version 12.5 (Agilent Technologies, SantaClara, CA, USA) selecting the Robust Multi-Array Average algorithm (RMA) to background-adjust, normalize, and log-transform signals intensity. As the log_2_ scale was used, a signal log_2_ ratio of 1.0 corresponds to a transcript level increase of two-fold change (2 FC) and −1.0 a lowering of two-fold change (−2 FC). A signal log_2_ ratio of zero indicates no change. Relative gene expression levels of each transcript were validated applying the unpaired *t*-test (*p* ≤ 0.01) and the Bonferroni multiple testing correction. Genes that displayed an expression level at least 1.5 fold different in the test sample versus control sample (*p* ≤ 0.01) were submitted both to functional classification, using the Gene Ontology (GO) annotations, and to Pathway analysis, employing the Panther expression analysis tools (http://pantherdb.org/) [14]. The enrichment of all pathways and functional classes associated to the differentially expressed genes compared to the distribution of genes included on the Affymetrix HG-U133A microarray was analyzed and *p* values ≤ 0.05, calculated by the binomial statistical test were considered as significant enrichment.

### 2.3. Protein-Protein Interaction (PPI) Network Construction and Network Clustering

The Search Tool for the Retrieval of Interacting Genes (STRING version 1.0; http://string-db.org/) is a web-based database which comprises experimental as well as predicted interaction informations and covers >1100 completely sequenced organisms [15]. DEGs were mapped to the STRING database to detect protein-protein interactions (PPI) pairs that were validated by experimental studies or retrieved by text mining and homology analysis [16]. A score of ≥0.7 for each PPI pair was selected to design the PPI network.

The graph-based Markov Clustering algorithm (MCL) allows to visualize high-flow areas (the clusters) [17]. In order to detect highly connected regions, the MCL algorithm was applied.

Cytoscape software [18] was used to define the topology of the builded networks.

### 2.4. Real Time RT-PCR

Total RNA was isolated from PBC using TRIzol reagent (Invitrogen, Carlsbad, CA, USA), following manufacturer’s instructions. First-strand cDNA was generated using the SuperScript III First-Strand Synthesis System for RT-PCR Kit (Invitrogen), with random hexamers, according to the manufacturer’s protocol. PCR was performed in a total volume of 25 μL containing 1× Taqman Universal PCR Master mix, no AmpErase UNG and 2.5 μL of cDNA; pre-designed, Gene-specific primers and probe sets for each gene (IL6ST, TNFRSF25, TNFSF8, and STAT1) were obtained from Assay-on-Demand Gene Expression Products (Applied Biosystems, Foster, CA, USA). As described in detail previously [19,20,21], Real Time PCR reactions were carried out in a two-tube system and in singleplex. The Real Time amplifications included 10 min at 95 °C (AmpliTaq Gold activation, Thermo Fisher Scientific, Waltham, MA, USA), followed by 40 cycles at 95 °C for 15 s and at 60 °C for one minute. Thermocycling and signal detection were performed with 7500 Sequence Detector (Applied Biosystems). Signals were detected according to the manufacturer’s instructions. This technique allows the identification of the cycling point where PCR product is detectable by means of fluorescence emission (Threshold cycle or *C*_t_ value). As previously reported, the *C*_t_ value correlates to the quantity of target mRNA [22]. Relative expression levels were calculated for each sample after normalization against the housekeeping genes GAPDH, beta-actin and 18 s ribosomal RNA (rRNA), using the ΔΔ*C*_t_ method for comparing relative fold expression differences [19,23]. The data are expressed as fold change. *C*_t_ values for each reaction were determined using TaqMan SDS analysis software. For each amount of RNA tested triplicate *C*_t_ values were averaged. Because *C*_t_ values vary linearly with the logarithm of the amount of RNA, this average represents a geometric mean.

## 3. Results

In order to identify gene expression profiles associated with AS, we compared the gene expression profiles of PBC samples obtained from 10 AS patients before treatment with ADA with 10 PBC samples obtained from healthy age and sex matched donors.

We found that 740 modulated transcripts satisfied the Bonferroni–corrected *p* value criterion (*p* ≤ 0.01) and the fold change criterion (FC ≥ |1.5|), showing robust and statistically significant variation between AS and healthy controls samples. In particular, 615 and 125 transcripts resulted to be over- and underexpressed respectively. The complete list of modulated genes can be found in Supplementary Materials Table S2.

The Gene Ontology analysis showed that a large number of the modulated transcripts can be ascribed to biological processes that may play a role in AS, including: cell proliferation, extracellular matrix organization, angiogenesis, immune response, inflammatory response, interleukin-6 production, ossification, bone remodeling and signal transduction. Among these processes we noticed that signal transduction and immune response were the most represented biological processes. We also observed that immune response encompassed several Th-17-related genes including CD28 molecule, CD3 molecule epsilon (CD3E) and gamma (CD3G) subunits, CD4 molecule, transforming growth factor beta 1 (TGFB1), interleukin 6 receptor (IL6R), interleukin 6 signal transducer (IL6ST).

The signal transduction functional class included genes that may be ascribed to different subcategories including: inflammation mediated by chemokine and cytokine signaling pathway, Jak-Stat signaling pathway, Wnt signaling pathway, mirogen-activated protein kinase (MAPK) cascade, tumor necrosis factor-mediated signaling pathway, TLR signaling pathway, cytokine-mediated signaling pathway, Type I interferon signaling pathway, fibroblast growth factor receptor signaling pathway, T-cell receptor signaling pathway, small GTPase mediated signal transduction, transforming growth factor (TGFB) signaling, Notch signaling and phosphatidylinositol-mediated signaling.

Table 1 and Table 2 show a detailed selection of DEGs within the above-mentioned processes. The tables also include GeneBank accession numbers and fold changes.

**Figure 1:**
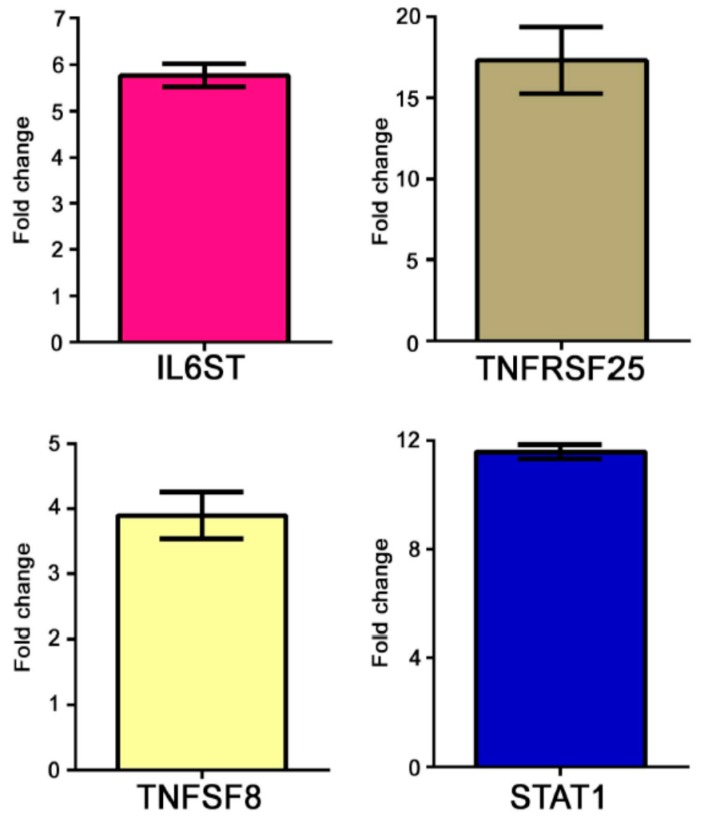
Real time (RT)-PCR of some modulated genes in AS patients. Genes selected for validation were IL6ST, TNFRSF25, TNFSF8 and STAT1. The transcripts of the selected genes were increased in AS samples when compared to healthy donors. Relative expression levels were calculated for each sample after normalization against the housekeeping gene GAPDH. Experiments have been conducted in triplicates. Similar results were obtained using the housekeeping genes18s rRNA and beta-actin.

The modulation of some genes observed by gene array analysis was validated by Q-PCR (Figure 1).

**Figure 2:**
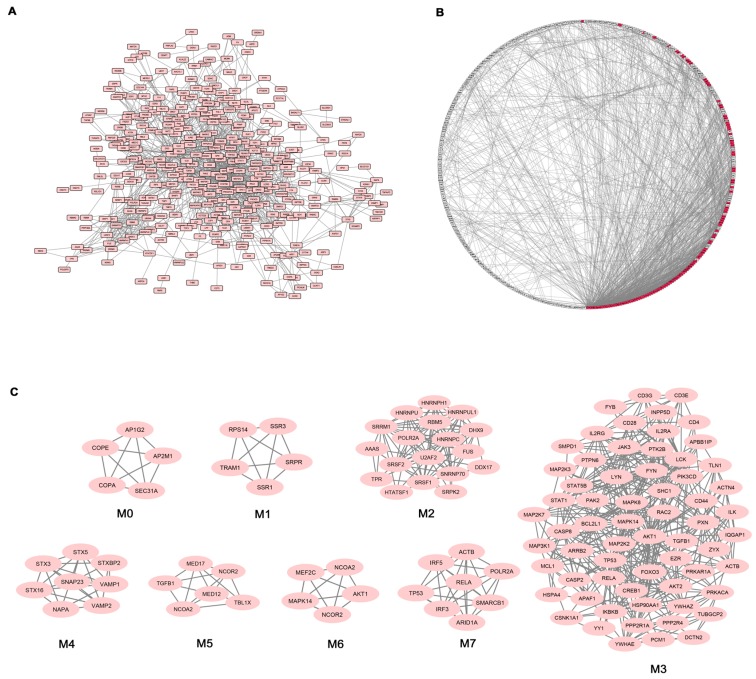
Network analysis of differentially expressed genes (DEGs) in AS patients. (**A**) Protein-protein interaction (PPI) network of DEGs; (**B**) Degree Sorted Circle Layout of the PPI network. Nodes are ordered around a circle based on their degree of connectivity (number of edges). Nodes belonging to modules are highlighted in red; (**C**) Modules originated from the interaction network.

Given the high number of DEGs and the corresponding functional classes, to make our study more informative, we decided to complement the gene expression profiling of AS PBC with the study of functional interactions between the protein products of modulated genes. To this aim, a protein-protein interaction (PPI) network comprising 401 genes (nodes) and 1345 pairs of interactions (edges) was obtained (Figure 2A). In Figure 2B the interconnected genes are represented in a circular graph and are ordered based on their degree of connectivity, so genes with a similar degree of connectivity are placed close to each other. We then submitted the PPI network to a modular analysis to find set of densely interconnected nodes (modules) that are expected to participate in multiple biological activities in a coordinated manner. We identified eight modules (*k* = 5) that are graphically represented in Figure 2C. Figure 2B shows that the genes belonging to the eight modules (highlighted in red) are distributed (concentrated) over an area in which the highest degrees of connectivity are present, thus confirming that they are highly connected genes.

We next performed functional enrichment analysis to identify association of genes, in each module, with different “GO terms” and pathways. All the significantly enriched categories for each module are shown in Supplementary Materials Table S3 and included pathogenetically relevant processes such as: T-cell receptor signaling (module M3), TLRs signaling (modules M3 and M7), small GTPase signaling (module M3) Wnt signaling (module M7), p38 MAP kinase cascade (modules M3 and M6) Jak-stat signaling (module M3) fibroblast growth factor receptor signaling (module M3) inflammation mediated by chemokine and cytokine signaling pathway (modules M3 and M7) VEGF signaling (modules M3 and M6) angiogenesis (module M3).

In order to evaluate the effect of treatment on gene expression in AS patients, we analysed the transcriptional profiles obtained from the same AS patients seven days after ADA administration.

**Figure 3:**
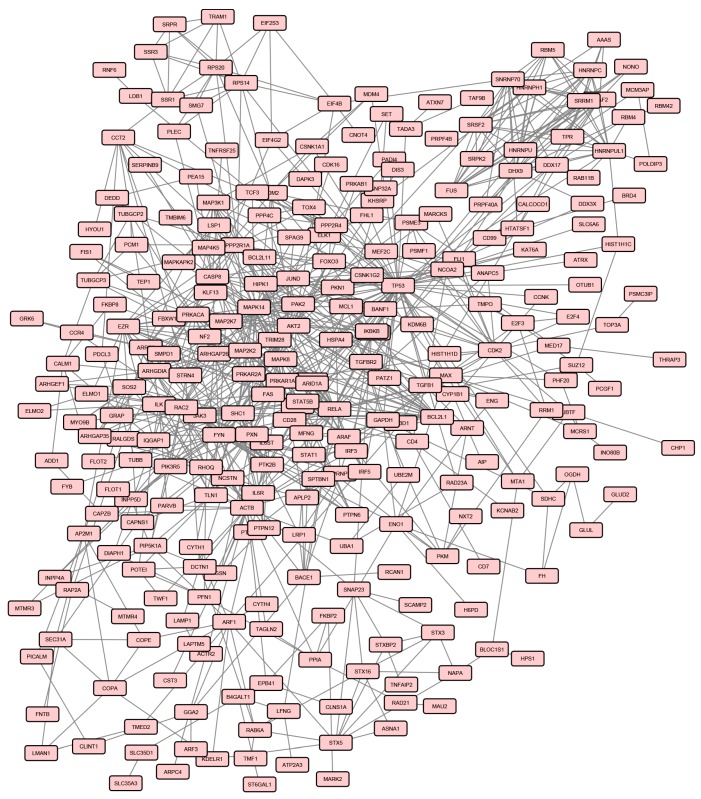
Protein-protein interaction (PPI) network of DEGs in AS patients after treatment.

When the same Bonferroni-corrected *p* value and fold change criteria were applied, 571 genes were differentially expressed in AS patients after treatment compared to healthy controls, in particular 492 and 79 transcripts resulted to be up- and down-regulated respectively (Supplementary Materials Table S4). The protein products of 297 DEGs, among the above mentioned genes, were connected in a PPI network involving 819 pairs of interactions (Figure 3), from which 7 modules can be extracted.

We next performed GO biological process and pathways enrichment analysis of genes included in the seven modules. Modules and the resulting enriched functional categories are listed in Supplementary Materials Table S5.

Afterwards, we compared the lists of processes enriched in modules from the two analysis (samples before treatment versus healthy controls and samples after treatment versus healthy controls) and we found that several pathways and biological processes were enriched only in the pre-treatment associated modules (Table 3).

In particular, we found that after treatment the enrichment in p38 MAPK (*p* value = 0.007), P53 pathway feedback loops 1 (*p* value = 0.005), PI3 kinase pathway (*p* value = 0.015) and Wnt signaling pathways (*p* value < 0.001) disappeared. In addition, we observed that all pathways that showed an enrichment only in the pre-treatment condition were associated to the pre-treatment associated modules M3, M6 and M7.

Many biological processes were also influenced by the therapy, since they were no longer enriched after treatment. These processes were associated to the pre-treatment module M3 and included: fibroblast growth factor receptor signaling (*p* value < 0.001), small GTPase mediated signal transduction (*p* value = 0.040), immune effector process (*p* value = 0.007), leukocyte migration (*p* value = 0.032), positive regulation of T-cell proliferation (*p* value = 0.035), response to interleukin-2 (*p* value = 0.001), T-cell costimulation (*p* value < 0.001), T-cell differentiation (*p* value = 0.015), T-cell receptor signaling (*p* value < 0.001), and phosphatidylinositol-mediated signaling pathway (*p* value = 0.002).

Although the patients enrolled in the study were homogeneous as far as clinical features and stage of the disease concern, we could identify two groups who showed a different response to treatment.

The first group (group 1) consisted of 6 patients who had a very good response to the treatment with disease remission and the second group (group 2) of four patients who showed a poor response to the treatment.

We then performed a gene array analysis by dividing patients in two groups, according to their response to the treatment, and comparing the expression profiles of two groups, before and after treatment, to those of the healthy control samples.

Before treatment we observed that in the first group of patients 689 genes, and in the second group 987 genes were modulated (Supplementary Materials Tables S6 and S7). After treatment, a modulation of 846 transcripts in the first group of patients and of 1077 genes in the second group was observed compared to the control group (Supplementary Materials Tables S8 and S9). When we compared genes modulated pre- and post-treatment in the two groups, we observed that 306 genes in the first group and 119 genes in the second group, returned to baseline expression levels after treatment, showing an expression level comparable to that of healthy controls.

To finely dissect the effect of ADA treatment on gene expression profiles in the two patients groups, we decided to functionally analyze these genes and we found that ADA had an impact on genes involved in signaling events that may be related to the pathogenesis of the disease. In particular, we compared the two lists of biological signaling in which DEGs, that were corrected by the treatment, were involved (Table 4).

We found that some of these categories were shared by the two groups of patients, (i.e., immune response, inflammatory response, angiogenesis, MAP kinase, Jak-Stat small GTPase, T-cell receptor, Notch and TGF-beta signaling) while others were only associated to group 1 (i.e., interleukin 6 production, cytokine mediated, NIK/NF-Kappa B, Toll-like receptor, Tumor necrosis factor, Type 1 interferon, and Wnt signaling) or to group 2 (i.e., EGF receptor, Ephrin receptor, Fc-gamma receptor signaling and positive regulation of BMP and VEGF signaling).

A selection of the above-mentioned genes, which were corrected by the treatment in the two groups of patients, referred to as “genes modulated only before treatment is reported in Table 5 and Table 6, where transcripts are grouped according to the Gene Ontology classification criteria. These tables also show the fold changes resulting from the comparison of pre- or post-treatment condition versus healthy controls.

Then we also performed a functional enrichment analysis to detect biological processes that were significantly enriched in genes corrected by the treatment.

Some of the enriched biological classes were related to the immune response and, in particular, to the activation of B and T-cell response, as indicated by the enrichment in T-cell activation (*p* value = 0.005), positive regulation of B-cell activation (*p* value = 0.041) and B-cell cytokine production (*p* value = 0.024) categories in the first group, and by the enrichment in T-cell activation (*p* value = 0.038) and positive regulation of B-cell activation (*p* value = 0.015) classes in the second group (Table 7). Genes involved in inflammation were also well represented in the two datasets modulated only before treatment and the two classes “inflammatory response” (*p* value = 0.026) and “NLRP3 inflammasome complex assembly” (*p* value = 0.006) were enriched in the first and in the second group, respectively.

In addition, we also identified functional classes that were enriched only in one of the two groups of patients. These classes were: positive regulation of cytokine production (*p* value = 0.012) positive regulation of type I interferon production (*p* value = 0.017) Toll-like receptor signaling pathway (*p* value = 0.039) positive regulation of interleukin-2 production (*p* value = 0.001) cellular response to unfolded protein (*p* value = 0.001) in the first group and, angiogenesis (*p* value = 0.002) response to transforming growth factor beta (*p* value = 0.010) and G-protein coupled receptor signaling pathway (*p* value = 0.031) in the second group.

## 4. Discussion

In this work, we first compared the gene expression profiles of 10 AS patients before treatment with 10 healthy controls. Secondly, we analysed the gene expression before and after ADA therapy, in order to identify biological processes that may be modulated by the drug. Finally, we compared gene profiles of patients characterized by a different response to the treatment.

Our results showed that AS has a profound impact on gene expression, since a large number of genes are modulated in AS samples. Moreover, DEGs have a role in biological processes that may be pathogenetically relevant to both the disease onset and progression.

The pathological features of AS are supposed to originate from several events that may interact, including biomechanical insult, genetic background, infections, inflammation and immune response, even though the precise mechanisms of the immune activation are still a matter of discussion [24].

We here identified a large number (40) of genes that have well-documented roles in the immune response, including for example TNFSF8/CD30L, interleukin-32 and several Th17-lymphocytes-related genes. TNFSF8 is a TNF-family member expressed on activated peripheral blood T-cells, B-cells, neutrophils, mast cells, monocytes, and macrophages and it is the ligand for CD30, a molecule that is present at high levels in AS patients compared to healthy controls [25]. The CD30/CD30L signaling system has been implicated in the pathogenesis of several autoimmune and inflammatory diseases and it has been recently associated to the pathogenesis of rheumatoid arthritis synovitis [26].

Interleukin-32 is a proinflammatory cytokine produced by T lymphocytes, natural killer cells, blood monocytes and fibroblast-like synoviocytes in the joints. It is present in the synovial fluid of AS patients at higher levels compared with RA or osteoarthritis patients and induces osteoblast differentiation leading to pathological bone growth [27].

Th17 cells have been associated to several autoimmune disorders including SLE, RA and psoriasis where they play a role in the pathogenesis of the disease [28,29,30]. Recent studies have demonstrated that Th17 cells are involved also in the pathogenesis of AS [31].

It is well known that the pathogenesis of AS is characterized by three phases: inflammation, erosion and abnormal bone outgrowth [32]. In this regard our data seem to recapitulate all these pathogenetic stages, showing deregulation of genes involved in the inflammatory process as well of transcripts that play a role in bone remodeling (i.e., bone growth and bone erosion).

Although the precise events that prime inflammation in spondyloarthritis is still unclear, recent findings provided evidence of the involvement of immune pathways, including the lately described IL-17/IL-23 pathway, NF-κB activation and antigen presentation [33] reintroducing the issue of autoimmune versus autoinflammatory disease. To date, however, no immunological pathway has emerged as the master regulator that may finely orchestrate the disease pathogenesis.

Interestingly, we found a remarkable number of DEGs included genes acting in commonly known immunological signalings such as TNF, MAP kinase cascade, Wnt, Toll-like receptor, TGF beta, T-cell receptor, Type I interferon, and Jak-stat signaling.

TNF-alpha is one of the main actors in the inflammatory scenario during the disease progression. Interestingly, here we show up-regulation of several genes involved in the TNF-alpha signaling including TNFRSF25. This molecule is a tumor necrosis factor receptor mainly expressed on T-cells [34] that has been associated with autoimmune diseases such as RA. Moreover, it has been reported that the absence of TNFRSF25 protects from the development of severe bone disorders in experimental antigen-induced arthritis (AIA) [35]. Along with the upregulation of TNF-related genes we also found overexpression of genes involved in interleukin-6 production and signal transduction (including IL6R and IL6ST) together with overexpression of transcripts of the MAP kinases cascade, two well-documented down-stream events of the biologic response to TNF [36]. In particular, it has been shown that high serum levels of IL-6 may associate with the development of AS [36]. The triggering of mitogen-activated kinase (MAPK) signaling cascade leads to activation of downstream JUN N-terminal kinase (JNK) and p38/MAPK14 [37], which is involved in the proinflammatory activity of interleukin 1 (IL-1) and TNF-alpha [38]. The transcript for this kinase was 1.8 fold increased in AS samples. Interestingly, it has been shown that inhibition of p38 can restore chondrocyte proliferation in inflammatory bone diseases such as spondyloarthritis [38].

The Wnt signaling components are critically involved in normal bone homeostasis, and in particular in new bone formation [32]. Moreover, it has been recently suggested that Wnt signaling may take part to the process of ankylosis in spondyloarthritis [32].

Deregulation of TLR signaling has been already described in AS [39] and there is emerging evidence of a potential role of TLRs in AS [40].

The up-regulation of genes involved in TGF-beta signaling is not surprising, since TGF-beta is important for bone formation and has been detected in biopsy specimens from sacroiliac joints of patient with AS where it may play a role in articular cartilage fibrosis and ossification [41]. Moreover, this cytokine is also involved in adaptive immune responses by regulating effector and regulatory CD4^+^ T-cell responses [42]. The importance of T-cell response is highlighted in our AS samples by the up-regulation of several T-cell related genes involved in T-cell receptor signaling including CD3g molecule, gamma (CD3G) and T-cell receptor alpha constant (TRAC). In particular, the association of this signaling pathway with AS has been already suggested by Chen et al. in a previous whole-blood gene expression study [43]. Among the modulated genes, we also found overexpression of transcripts for members of type I IFN signaling, a network typically associated with autoimmune diseases such as SLE, RA, Crohn’s disease and Sjogren syndrome [44,45,46,47,48,49,50].

In this regard, the co-presence of type I IFN signaling and Th-17 related genes may be suggestive for an autoimmune origin of AS, since the co-activity of IFN and Th17 pathways is typical of autoimmunity and has been described both in human diseases and in animal models [51,52,53].

Another signaling pathway relevant to autoimmunity is the Jak-stat pathway [54] that is represented in our dataset by the upregulation of STAT1 and STAT5b. It has been described that STAT1 gain-of-function mutations are associated to autoimmune response [54] and that this molecule is differentially expressed in AS patients by transcriptome network analysis [55]. In addition we also found modulation of genes involved in fibroblast growth factor receptor, small GTPase and Notch signalings. It is worthwhile mentioning that the first favours the formation of new bone in AS patients [56], the second triggers proliferation of rheumatoid synovial fibroblasts [57] and the Notch signaling is involved in ligament ossification of hip joints in AS [58].

In the next step of our data analysis we found that DEG products were connected in a wide PPI network from which we obtained eight functional modules, containing the most functionally interconnected genes. These modules were significantly enriched in several of the above mentioned pathologically relevant processes and pathways including: T-cell receptor signaling, TLRs signaling, small GTPase signaling, Wnt signaling, p38 MAP kinase cascade and Jak-stat signaling.

Moreover, this analysis was paralleled by the comparison of post-treatment samples to healthy controls and, in this case, the resulted PPI network included seven modules.

Interestingly, by comparing pathways and biological processes enrichments of modules derived from the pre- and the post-treatment conditions, we identified three pre-treatment-associated modules (M3, M6, and M7) towards which the drug seemed to direct its activity. Indeed several crucial processes and pathways, enriched in these modules, were no longer enriched after treatment, including p38 MAP kinase, Wnt, FGF receptor, small GTPase, T-cell receptor and PI3 kinase signaling.

To more finely dissect the impact of treatment on expression profiles of AS samples, we separately analyzed the two groups of patients that were characterized by a different response to the treatment.

The two analyses showed that a quite different number of genes were modulated before treatment in the two datasets. In particular, after comparing genes modulated before and after treatment in the two groups of patients, we found a different number of genes that returned to a normal expression level after treatment.

Focusing our analysis on these genes, we realized that globally, in both groups of patients, the treatment had an impact on many pathologically relevant biological processes, including immune response, inflammatory response, angiogenesis and on several signaling pathways (i.e., Jak-Stat signaling, MAP kinase cascade and Small GTPase signaling). However we also observed that only in the responders group (group 1), genes involved in Toll-like receptor signaling, tumor necrosis factor signaling, Type I interferon signaling and Wnt signaling returned to normal.

Moreover, when we specifically analyzed the categories enriched in genes corrected by the treatment in the two groups of patients, we observed, in both data sets, an enrichment in the inflammatory response and in the lymphocytes activation processes, but we also observed that several categories were enriched only in the group 1, including regulation of interleukin-2 production, cellular response to unfolded protein, positive regulation of type I interferon production and toll-like receptor signaling.

These results suggest that the differences emerged from the separated analysis of the two groups of patients, may be partially related to the different response to treatment. In particular, we have identified altered processes and signaling that may be more strongly associated to AS pathogenesis since they are corrected by the treatment in patients with a good response.

We suggest that these biological processes may be the target of novel therapies if our results are confirmed in further experiments and in a larger number of patients.

## 5. Conclusions

The etiology of AS is still unknown and the identification of the involved molecular pathogenetic pathways is a crucial issue in medicine. ADA in an humanized anti-TNF-alpha monoclonal antibody, used in the treatment of AS. Our study aimed at analyzing gene transcriptional profiles in patients with AS before and after treatment with ADA. A sophisticated analysis (i.e., PPI network clustering analysis) was used to study DEGs in AS patients before and after treatment.

By PPI network analysis it was possible to identify clusters of genes specifically associated to AS. Within these clusters DEGs belong to well known molecular pathways which play a prominent role in disease pathogenesis. Some of these pathways are strongly modified by ADA in patients with a good response to the treatment. These potentially pathogenetic pathways are not significantly modified in non responder subjects. Clustering network analysis is therefore a promising tool in gene expression studies and may help to identify new potential therapeutical targets in AS.

## Figures and Tables

**Table 1:** Genes modulated in Ankylosing spondylitis (AS) patients before treatment versus healthy controls.

Probe Set ID	*p* Value	Fold Change	Gene Symbol	Gene Title	Representative Public ID
**Cell proliferation**					
201367_s_at	0.001	13.9	ZFP36L2	ZFP36 ring finger protein-like 2	NM_006887
203055_s_at	0.006	2.1	ARHGEF1	Rho guanine nucleotide exchange factor (GEF) 1	NM_004706
210541_s_at	0.014	1.7	TRIM27	tripartite motif containing 27	AF230394
217202_s_at	0.011	2.4	GLUL	glutamate-ammonia ligase	NM_002065
201234_at	0.001	2.0	ILK	integrin-linked kinase	NM_004517
210440_s_at	0.013	1.6	CDC14A	cell division cycle 14A	AF064102
**Extracellular matrix organization**					
202524_s_at	0.013	1.6	SPOCK2	sparc/osteonectin, cwcv and kazal-like domains proteoglycan (testican) 2	NM_001134434
205179_s_at	0.002	2.3	ADAM8	ADAM metallopeptidase domain 8	NM_001109
216971_s_at	0.001	2.5	PLEC	plectin	Z54367
216627_s_at	0.004	2.6	B4GALT1	UDP-Gal:betaGlcNAc beta 1,4-galactosyltransferase, polypeptide 1	U10473
200001_at	0.013	1.9	CAPNS1	calpain, small subunit 1	NM_001749
**Angiogenesis**					
202509_s_at	<0.001	1.6	TNFAIP2	tumor necrosis factor, alpha-induced protein 2	XM_006720243
201559_s_at	0.013	1.8	CLIC4	chloride intracellular channel 4	CR533501
214931_s_at	0.002	1.6	SRPK2	SRSF protein kinase 2	NM_001278273
202434_s_at	0.003	1.8	CYP1B1	cytochrome P450, family 1, subfamily B, polypeptide 1	NM_000104
205179_s_at	0.002	2.3	ADAM8	ADAM metallopeptidase domain 8	NM_001109
**Immune response**					
207216_at	0.003	1.7	TNFSF8	tumor necrosis factor (ligand) superfamily, member 8	NM_001244
214971_s_at	0.000	4.3	ST6GAL1	ST6 beta-galactosamide alpha-2,6-sialyltranferase 1	NM_173216
216901_s_at	0.000	13.6	IKZF1	IKAROS family zinc finger 1 (Ikaros)	NM_006060
211861_x_at	0.002	3.5	CD28	CD28 molecule	AF222343
205456_at	0.011	3.0	CD3E	CD3e molecule, epsilon (CD3-TCR complex)	NM_000733
214551_s_at	0.009	3.5	CD7	CD7 molecule	NM_006137
205831_at	0.012	1.9	CD2	CD2 molecule	NM_001767
207872_s_at	0.002	2.0	LILRA1	leukocyte immunoglobulin-like receptor, subfamily A, member 1	NM_006863
208594_x_at	0.015	1.8	LILRA6	leukocyte immunoglobulin-like receptor, subfamily A, member 6	NM_024318
205819_at	0.003	−1.8	MARCO	macrophage receptor with collagenous structure	NM_006770
215339_at	0.007	1.9	NKTR	natural killer-tumor recognition sequence	NM_005385
208829_at	0.008	1.5	TAPBP	TAP binding protein (tapasin)	AF029750
209754_s_at	<0.001	3.4	TMPO	thymopoietin	NM_003276
206804_at	0.005	1.8	CD3G	CD3g molecule, gamma (CD3-TCR complex)	NM_000073
203547_at	0.008	2.4	CD4	CD4 molecule	NM_000616
205468_s_at	0.002	2.4	IRF5	interferon regulatory factor 5	NM_001098627
203227_s_at	0.011	−1.5	TSPAN31	tetraspanin 31	NM_005981
202621_at	0.002	2.2	IRF3	interferon regulatory factor 3	NM_001571
210370_s_at	0.005	1.7	LY9	lymphocyte antigen 9	AF244129
214931_s_at	0.002	1.6	SRPK2	SRSF protein kinase 2	NM_001278273
216208_s_at	0.004	1.8	ATF6B	activating transcription factor 6 beta	NM_004381
211574_s_at	<0.001	2.5	CD46	CD46 molecule, complement regulatory protein	D84105
211192_s_at	0.001	2.8	CD84	CD84 molecule	AF054818
210850_s_at	0.006	1.6	ELK1	ELK1, member of ETS oncogene family	AF000672
210313_at	0.012	−1.7	LILRA4	leukocyte immunoglobulin-like receptor, subfamily A, member 4	NM_012276
200887_s_at	<0.001	4.6	STAT1	signal transducer and activator of transcription 1	GU211347
204770_at	0.009	1.8	TAP2	transporter 2, ATP-binding cassette	NM_000544
206409_at	0.007	1.8	TIAM1	T-cell lymphoma invasion and metastasis 1	NM_003253
208602_x_at	<0.001	3.8	CD6	CD6 molecule	NM_006725
220132_s_at	0.004	2.8	CLEC2D	C-type lectin domain family 2, member D	NM_013269
201835_s_at	<0.001	1.8	PRKAB1	protein kinase, AMP-activated, beta 1	NM_006253
204312_x_at	0.009	1.6	CREB1	cAMP responsive element binding protein 1	NM_004379
221602_s_at	0.006	2.8	FAIM	Fas apoptotic inhibitory molecule	NM_001033030
201234_at	0.001	2.0	ILK	integrin-linked kinase	NM_004517
210796_x_at	0.010	1.6	SIGLEC6	sialic acid binding Ig-like lectin 6	D86359
205026_at	0.001	1.8	STAT5B	signal transducer and activator of transcription 5B	NM_012448
210298_x_at	0.010	1.8	FHL1	four and a half LIM domains 1	AF098518
204864_s_at	0.003	3.2	IL6ST	interleukin 6 signal transducer	NM_002184
201246_s_at	0.004	1.8	OTUB1	OTU domain, ubiquitin aldehyde binding 1	NM_017670
203828_s_at	0.001	3.2	IL32	interleukin 32	NM_004221
**Inflammatory response**					
208540_x_at	0.011	2.0	S100A11P1	S100 calcium binding protein A11 pseudogene 1	NG_008400
211968_s_at	0.004	1.6	HSP90AA1	heat shock protein 90kDa alpha (cytosolic), class A member 1	NM_001017963
211016_x_at	0.000	2.8	HSPA4	heat shock 70kDa protein 4	BC002526
200806_s_at	0.001	2.2	HSPD1	heat shock 60kDa protein 1 (chaperonin)	KU178141
202626_s_at	0.004	−1.9	LYN	v-yes-1 Yamaguchi sarcoma viral related oncogene homolog	NM_002350
203085_s_at	0.005	2.5	TGFB1	transforming growth factor, beta 1	NM_000660
203899_s_at	0.001	1.6	CRCP	CGRP receptor component	NM_014478
205179_s_at	0.002	2.3	ADAM8	ADAM metallopeptidase domain 8	NM_001109
207163_s_at	0.007	1.6	AKT1	v-akt murine thymoma viral oncogene homolog 1	XM_017021075
210916_s_at	0.013	1.9	CD44	CD44 molecule (Indian blood group)	AF098641
206341_at	0.012	1.6	IL2RA	interleukin 2 receptor, alpha	NM_000417
208376_at	0.004	1.6	CCR4	chemokine (C-C motif) receptor 4	NM_005508
211027_s_at	0.001	3.4	IKBKB	inhibitor of kappa light polypeptide gene enhancer in B-cells, kinase beta	NM_001190720
217489_s_at	0.002	2.6	IL6R	interleukin 6 receptor	S72848
**Interleukin-6 production**					
200806_s_at	0.001	2.2	HSPD1	heat shock 60kDa protein 1 (chaperonin)	KU178141
203331_s_at	0.005	1.6	INPP5D	inositol polyphosphate-5-phosphatase, 145kDa	U53470
217489_s_at	0.002	2.6	IL6R	interleukin 6 receptor	S72848
201461_s_at	0.002	2.4	MAPKAPK2	mitogen-activated protein kinase-activated protein kinase 2	NM_004759
203388_at	0.011	2.3	ARRB2	arrestin, beta 2	NM_004313
**Bone remodeling**					
**(a) bone growth**					
203085_s_at	0.005	2.52	TGFB1	transforming growth factor, beta 1	BC000125
205485_at	0.013	1.53	RYR1	ryanodine receptor 1 (skeletal)	NM_000540
206788_s_at	0.003	1.72	CBFB	core-binding factor, beta subunit	AF294326
207163_s_at	0.007	1.55	AKT1	v-akt murine thymoma viral oncogene homolog 1	XM_017021075
210671_x_at	0.006	1.70	MAPK8	mitogen-activated protein kinase 8	NM_001278548
214326_x_at	0.007	3.88	JUND	jun D proto-oncogene	NM_005354
212105_s_at	0.000	4.8	DHX9	DEAH (Asp-Glu-Ala-His) box helicase 9	NM_001357
207968_s_at	0.003	2.12	MEF2C	myocyte enhancer factor 2C	NM_002397
206788_s_at	0.003	1.72	CBFB	core-binding factor, beta subunit	AF294326
216234_s_at	0.004	1.79	PRKACA	protein kinase, cAMP-dependent, catalytic, alpha	M80335
**(b) bone erosion**					
214196_s_at	<0.001	2.41	TPP1	tripeptidyl peptidase I	NM_000391
203331_s_at	0.005	1.57	INPP5D	inositol polyphosphate-5-phosphatase, 145 kDa	U53470
205179_s_at	0.002	2.31	ADAM8	ADAM metallopeptidase domain 8	NM_001109
203395_s_at	0.002	−1.97	HES1	hairy and enhancer of split 1	NM_005524

**Table 2:** Genes modulated in AS patients before treatment versus healthy controls grouped in signaling pathways.

Probe Set ID	*p* Value	Fold Change	Gene Symbol	Gene Title	Representative Public ID
**Jak-Stat signaling pathway**					
200887_s_at	<0.001	4.61	STAT1	signal transducer and activator of transcription 1, 91 kDa	GU211347
205026_at	0.001	1.82	STAT5B	signal transducer and activator of transcription 5B	NM_012448
**Wnt signaling pathway**					
214279_s_at	0.011	1.89	NDRG2	NDRG family member 2	NM_201535
217729_s_at	0.004	2.29	AES	amino-terminal enhancer of split	NM_001130
209456_s_at	0.003	2.03	FBXW11	F-box and WD repeat domain containing 11	AB033281
209002_s_at	0.004	1.94	CALCOCO1	calcium binding and coiled-coil domain 1	NM_020898
206562_s_at	0.003	1.53	CSNK1A1	casein kinase 1, alpha 1	NM_001892
205254_x_at	0.007	2.57	TCF7	transcription factor 7 (T-cell specific, HMG-box)	NM_003202
**MAPK cascade**					
210671_x_at	0.006	1.70	MAPK8	mitogen-activated protein kinase 8	NM_001278548
209952_s_at	0.001	1.70	MAP2K7	mitogen-activated protein kinase kinase 7	AF006689
214786_at	0.001	1.96	MAP3K1	mitogen-activated protein kinase kinase kinase 1	NM_005921
211081_s_at	0.004	2.44	MAP4K5	mitogen-activated protein kinase kinase kinase kinase 5	Z25426
201461_s_at	0.002	2.38	MAPKAPK2	mitogen-activated protein kinase-activated protein kinase 2	NM_004759
210449_x_at	0.005	1.79	MAPK14	mitogen-activated protein kinase 14	NM_001315
201979_s_at	0.009	1.64	PPP5C	protein phosphatase 5, catalytic subunit	NM_006247
213490_s_at	0.001	2.49	MAP2K2	mitogen-activated protein kinase kinase 2	NM_030662
207968_s_at	0.003	2.12	MEF2C	myocyte enhancer factor 2C	NM_002397
215220_s_at	0.003	5.73	TPR	translocated promoter region, nuclear basket protein	NM_003292
**Tumor necrosis factor-mediated signaling**					
211282_x_at	0.014	2.20	TNFRSF25	tumor necrosis factor receptor superfamily, member 25	AY254324
209852_x_at	0.003	1.69	PSME3	proteasome (prosome, macropain) activator subunit 3	BC001423
200887_s_at	0.000	4.61	STAT1	signal transducer and activator of transcription 1, 91 kDa	GU211347
211027_s_at	0.001	3.41	IKBKB	inhibitor of kappa light polypeptide gene enhancer in B-cells	NM_001190720
**TLR signaling pathway**					
210449_x_at	0.005	1.79	MAPK14	mitogen-activated protein kinase 14	NM_001315
211027_s_at	0.001	3.41	IKBKB	inhibitor of kappa light polypeptide gene enhancer in B-cells	NM_001190720
215499_at	0.001	−1.55	MAP2K3	mitogen-activated protein kinase kinase 3	GQ225578
210671_x_at	0.006	1.70	MAPK8	mitogen-activated protein kinase 8	NM_001278548
209878_s_at	0.002	1.56	RELA	v-rel reticuloendotheliosis viral oncogene homolog A	M62399
**Type I interferon signaling pathway**					
205468_s_at	0.002	2.41	IRF5	interferon regulatory factor 5	NM_001098627
202621_at	0.002	2.15	IRF3	interferon regulatory factor 3	NM_001571
200887_s_at	<0.001	4.61	STAT1	signal transducer and activator of transcription 1, 91 kDa	GU211347
211209_x_at	0.004	1.60	SH2D1A	SH2 domain containing 1A	AF100540
**Fibroblast growth factor receptor signaling**					
213490_s_at	0.001	2.49	MAP2K2	mitogen-activated protein kinase kinase 2	NM_030662
210655_s_at	0.001	1.78	FOXO3	forkhead box O3	AF041336
216234_s_at	0.004	1.79	PRKACA	protein kinase, cAMP-dependent, catalytic, alpha	M80335
213472_at	0.000	5.14	HNRNPH1	heterogeneous nuclear ribonucleoprotein H1 (H)	NM_001257293
**T-cell receptor signaling**					
207485_x_at	0.004	1.65	BTN3A1	butyrophilin, subfamily 3, member A1	NM_007048
205285_s_at	0.001	3.30	FYB	FYN binding protein	NM_001465
204890_s_at	0.010	1.69	LCK	lymphocyte-specific protein tyrosine kinase	U07236
206804_at	0.005	1.83	CD3G	CD3g molecule, gamma (CD3-TCR complex)	NM_000073
209671_x_at	0.012	2.31	TRAC	T-cell receptor alpha constant	M12423
**Small GTPase mediated signal transduction**					
201469_s_at	0.002	1.82	SHC1	Src homology 2 domain containing transforming protein 1	X68148
213490_s_at	0.001	2.49	MAP2K2	mitogen-activated protein kinase kinase 2	NM_030662
207419_s_at	<0.001	2.45	RAC2	ras-related C3 botulinum toxin substrate 2	NM_002872
208876_s_at	0.001	1.81	PAK2	p21 protein (Cdc42/Rac)-activated kinase 2	AF092132
206341_at	0.012	1.58	IL2RA	interleukin 2 receptor, alpha	NM_000417
204116_at	0.004	2.49	IL2RG	interleukin 2 receptor, gamma	NM_000206
210317_s_at	0.001	2.71	YWHAE	tyrosine 3-monooxygenase	U28936
X00351_5_at	0.003	2.65	ACTB	actin, beta	NM_001101
211108_s_at	0.009	1.86	JAK3	Janus kinase 3	U31601
200641_s_at	<0.001	1.83	YWHAZ	tyrosine 3-monooxygenase	NM_003406
216033_s_at	0.001	1.87	FYN	FYN oncogene related to SRC, FGR, YES	NM_002037
**Transforming growth factor beta signaling**					
203085_s_at	0.005	2.52	TGFB1	transforming growth factor, beta 1	NM_000660
207334_s_at	0.000	2.69	TGFBR2	transforming growth factor, beta receptor II (70/80 kDa)	NM_003242
219543_at	0.012	−1.75	PBLD	phenazine biosynthesis-like protein domain containing	NM_022129
211553_x_at	0.013	1.70	APAF1	apoptotic peptidase activating factor 1	AF149794
200808_s_at	0.004	2.60	ZYX	zyxin	NM_003461
204312_x_at	0.009	1.62	CREB1	cAMP responsive element binding protein 1	NM_004379
**Phosphatidylinositol-mediated signaling**					
217234_s_at	0.003	2.51	EZR	ezrin	NM_003379
211230_s_at	0.005	1.60	PIK3CD	phosphatidylinositol-4,5-bisphosphate 3-kinase	U57843
204890_s_at	0.010	1.69	LCK	lymphocyte-specific protein tyrosine kinase	U07236
**Notch signaling**					
215270_at	0.007	1.85	LFNG	LFNG O-fucosylpeptide 3-beta-*N*-acetylglucosaminyltransferase	U94354
204152_s_at	0.005	1.94	MFNG	MFNG O-fucosylpeptide 3-beta-*N*-acetylglucosaminyltransferase	NM_002405.3
206341_at	0.012	1.58	IL2RA	interleukin 2 receptor, alpha	NM_000417

**Table 3:** Pathways and biological processes that were enriched only pre- or post treatment.

**Enriched Pathways**			
**Pre-Treatment vs. Healthy Controls**	***p*** **Value**	**Post-Treatment vs. Healthy Controls**	***p*** **Value**
Insulin/IGF pathway-protein kinase B signaling cascade	0.005	FAS signaling pathway	0.007
Oxidative stress response	0.001	Histamine H2 receptor mediated signaling pathway	0.003
p38 MAPK pathway	0.007	p53 pathway feedback loops 2	0.022
p53 pathway	0.008	Transcription regulation by bZIP transcription factor	0.042
p53 pathway by glucose deprivation	0.014	Heterotrimeric G-protein signaling pathway	0.005
P53 pathway feedback loops 1	0.005		
PI3 kinase pathway	0.015		
Wnt signaling pathway	<0.001		
**Enriched Biological Processes**			
**Pre-Treatment vs. Healthy Controls**	***p*** **Value**	**Post-Treatment vs. Healthy Controls**	***p*** **Value**
ATP-dependent chromatin remodeling	0.025	activation of protein kinase A activity	<0.001
cytoskeleton organization	0.002	cellular response to oxidative stress	0.010
fibroblast growth factor receptor signaling pathway	<0.001	extrinsic apoptotic signaling pathway in absence of ligand	0.007
immune effector process	0.007	hematopoietic or lymphoid organ development	0.037
intrinsic apoptotic signaling pathway	<0.001	negative regulation of extrinsic apoptotic signaling pathway	<0.001
leukocyte migration	0.032	peptidyl-serine phosphorylation	0.001
negative regulation of protein phosphorylation	0.048	peptidyl-tyrosine phosphorylation	<0.001
phosphatidylinositol-mediated signaling	0.002	protein autophosphorylation	0.010
positive regulation of cell differentiation	0.004	Ras protein signal transduction	0.001
positive regulation of gene expression	0.019	regulation of lymphocyte activation	0.026
positive regulation of interleukin-2 biosynthetic process	0.003	regulation of mitochondrial membrane permeability	0.039
positive regulation of T-cell proliferation	0.035	regulation of myeloid cell differentiation	0.004
regulation of execution phase of apoptosis	0.045		
regulation of leukocyte mediated immunity	0.005		
regulation of lymphocyte differentiation	0.017		
response to interleukin-2	0.001		
response to mechanical stimulus	0.004		
small GTPase mediated signal transduction	0.040		
T-cell costimulation	<0.001		
T-cell differentiation	0.015		
T-cell homeostasis	0.004		
T-cell receptor signaling pathway	<0.001		

**Table 4:** Molecular signalings in which are involved genes modulated only before treatment.

Patients Group 1	Patients Group 2
Immune response	Immune response
Angiogenesis	Angiogenesis
Inflammation	Inflammation
G-protein coupled receptor signaling	G-protein coupled receptor signaling
Jak-Stat signaling	Jak-Stat signaling
MAP kinase cascade	MAP kinase cascade
Notch signaling	Notch signaling
PDGF receptor signaling	PDGF receptor signaling
Small GTPase signaling	Small GTPase signaling
T-cell receptor signaling	T-cell receptor signaling
TGF beta signaling	TGF beta signaling
Intreleukin 6 production	EGF receptor signaling
Adenylate cyclase-modulating G-protein coupled receptor signaling	Ephrin receptor signaling
Chemokine mediated signaling	Fc-gamma receptor signaling
Cytokine mediated signaling	Integrin-mediated signaling pathway
Endothelin signaling	Interleukin signaling
Heterotrimeric G-protein signaling	Positive regulation of BMP signaling
Intrinsic apoptotic signaling in response to DNA damage signaling	Positive regulation of VEGF signaling
NIK/NF-Kappa B signaling	
Protein kinase B signaling	
Stimulatory c-type lectin receptor signaling	
Toll-like receptor signaling	
Tumor necrosis factor signaling	
Type 1 interferon signaling	
Wnt signaling	

**Table 5:** Genes modulated only before treatment in AS patients group 1. n.c.: not changed.

Probe Set ID	*p* Value	FC Pre	FC Post	Gene Symbol	Gene Title	Representative Public ID
**Immune response**						
207794_at	0.015	5.05	n.c.	CCR2	chemokine (C-C motif) receptor 2	NM_000648
216033_s_at	0.013	1.70	n.c.	FYN	FYN proto-oncogene, Src family tyrosine kinase	S74774
208965_s_at	0.003	2.87	n.c.	IFI16	interferon, gamma-inducible protein 16	BG256677
200806_s_at	0.004	2.62	n.c.	HSPD1	heat shock 60kDa protein 1 (chaperonin)	BE256479
211210_x_at	0.015	1.53	n.c.	SH2D1A	SH2 domain containing 1A	AF100539
210541_s_at	<0.001	2.21	n.c.	TRIM27	tripartite motif containing 27	AF230394
221092_at	0.007	4.53	n.c.	IKZF3	IKAROS family zinc finger 3 (Aiolos)	NM_012481
202643_s_at	0.003	−4.55	n.c.	TNFAIP3	tumor necrosis factor, alpha-induced protein 3	AI738896
219994_at	0.009	1.72	n.c.	APBB1IP	amyloid beta precursor protein-binding, B, 1 interacting protein	NM_019043
220363_s_at	0.012	1.79	n.c.	ELMO2	engulfment and cell motility 2	NM_022086
204506_at	0.009	1.50	n.c.	PPP3R1	protein phosphatase 3, regulatory subunit B, alpha	AL544951
**Inflammatory response**						
201883_s_at	0.001	−2.05	n.c.	B4GALT1	beta 1,4- galactosyltransferase, polypeptide 1	D29805
207794_at	0.015	5.05	n.c.	CCR2	chemokine (C-C motif) receptor 2	NM_000648
214370_at	0.007	1.91	n.c.	S100A8	S100 calcium binding protein A8	AW238654
200808_s_at	0.002	2.02	n.c.	ZYX	zyxin	NM_003461
210218_s_at	0.002	2.50	n.c.	SP100	SP100 nuclear antigen	U36501
205760_s_at	0.011	1.62	n.c.	OGG1	8-oxoguanine DNA glycosylase	NM_016821
209545_s_at	0.011	−1.95	n.c.	RIPK2	receptor-interacting serine-threonine kinase 2	AF027706
202742_s_at	0.011	1.91	n.c.	PRKACB	protein kinase, cAMP-dependent, catalytic, beta	NM_002731
204280_at	0.005	1.53	n.c.	RGS14	regulator of G-protein signaling 14	NM_006480
203927_at	0.003	−1.83	n.c.	NFKBIE	nuclear factor of k light polypeptide gene enhancer in B-cells inhibitor, epsilon	NM_004556
205727_at	0.015	−1.68	n.c.	TEP1	telomerase-associated protein 1	NM_007110
**Angiogenesis**						
212723_at	0.012	−2.14	n.c.	JMJD6	jumonji domain containing 6	AK021780
213844_at	0.014	−2.13	n.c.	HOXA5	homeobox A5	NM_019102
211561_x_at	0.006	1.91	n.c.	MAPK14	mitogen-activated protein kinase 14	L35253
201883_s_at	0.001	−2.05	n.c.	B4GALT1	beta 1,4- galactosyltransferase, polypeptide 1	D29805
**TNF signaling pathway**						
207794_at	0.015	5.05	n.c.	CCR2	chemokine (C-C motif) receptor 2	NM_000648
222142_at	0.011	−1.82	n.c.	CYLD	cylindromatosis (turban tumor syndrome)	AK024212
202643_s_at	0.003	−4.55	n.c.	TNFAIP3	tumor necrosis factor, alpha-induced protein 3	AI738896
**Type 1 interferon signaling**						
210218_s_at	0.002	2.50	n.c.	SP100	SP100 nuclear antigen	U36501
208965_s_at	0.003	2.87	n.c.	IFI16	interferon, gamma-inducible protein 16	BG256677
200806_s_at	0.004	2.62	n.c.	HSPD1	heat shock 60kDa protein 1 (chaperonin)	BE256479
222142_at	0.011	−1.82	n.c.	CYLD	cylindromatosis (turban tumor syndrome)	AK024212
202643_s_at	0.003	−4.55	n.c.	TNFAIP3	tumor necrosis factor, alpha-induced protein 3	AI738896
221287_at	0.010	1.50		RNASEL	ribonuclease L (2′,5′-oligoisoadenylate synthetase-dependent)	NM_021133
**IL6 production**						
207794_at	0.015	5.05	n.c.	CCR2	chemokine (C-C motif) receptor 2	NM_000648
200806_s_at	0.004	2.62	n.c.	HSPD1	heat shock 60kDa protein 1 (chaperonin)	BE256479
**Toll like receptor signaling pathway**						
200598_s_at	0.011	1.54	n.c.	HSP90B1	heat shock protein 90kDa beta (Grp94), member 1	AI582238
200806_s_at	0.004	2.62	n.c.	HSPD1	heat shock 60kDa protein 1 (chaperonin)	BE256479
211561_x_at	0.006	1.91	n.c.	MAPK14	mitogen-activated protein kinase 14	L35253
**Jak-Stat signaling pathway**						
205026_at	0.005	1.85	n.c.	STAT5B	signal transducer and activator of transcription 5B	NM_012448
211561_x_at	0.006	1.91	n.c.	MAPK14	mitogen-activated protein kinase 14	L35253
**Wnt signaling pathway**						
208074_s_at	0.008	1.50	n.c.	AP2S1	adaptor-related protein complex 2, sigma 1 subunit	NM_021575
204506_at	0.009	1.50	n.c.	PPP3R1	protein phosphatase 3, regulatory subunit B, alpha	AL544951
201868_s_at	0.012	2.29	n.c.	TBL1X	transducin (beta)-like 1X-linked	NM_005647
203506_s_at	0.014	1.80	n.c.	MED12	mediator complex subunit 12	NM_005120
209383_at	0.006	−2.32	n.c.	DDIT3	DNA-damage-inducible transcript 3	BC003637
213710_s_at	0.014	1.59	n.c.	CALM1	calmodulin 1 (phosphorylase kinase, delta)	AL523275
206409_at	0.009	1.99	n.c.	TIAM1	T-cell lymphoma invasion and metastasis 1	NM_003253
213579_s_at	0.008	−1.51	n.c.	EP300	E1A binding protein p300	AI459462
210649_s_at	0.004	2.81	n.c.	ARID1A	AT rich interactive domain 1A (SWI-like)	AF231056
211300_s_at	0.009	3.08	n.c.	TP53	tumor protein p53	K03199
**MAPK cascade**						
210622_x_at	0.012	1.63	n.c.	CDK10	cyclin-dependent kinase 10	AF153430
213710_s_at	0.014	1.59	n.c.	CALM1	calmodulin 1 (phosphorylase kinase, delta)	AL523275
209457_at	0.013	−2.95	n.c.	DUSP5	dual specificity phosphatase 5	U16996
211561_x_at	0.006	1.91	n.c.	MAPK14	mitogen-activated protein kinase 14	L35253
216033_s_at	0.013	1.70	n.c.	FYN	FYN proto-oncogene, Src family tyrosine kinase	S74774
**Small Gtpase mediated signal transduction**						
205461_at	<0.001	2.98	n.c.	RAB35	RAB35, member RAS oncogene family	NM_006861
219842_at	0.011	1.50	n.c.	ARL15	ADP-ribosylation factor-like 15	NM_019087
209051_s_at	0.001	1.58	n.c.	RALGDS	ral guanine nucleotide dissociation stimulator	AF295773
211622_s_at	0.001	1.80	n.c.	ARF3	ADP-ribosylation factor 3	M33384

**Table 6:** Genes modulated only before treatment in AS patients-group 2. n.c.: not changed.

Probe Set ID	*p* Value	FC Pre	FC Post	Gene Symbol	Gene Title	Representative Public ID
**Immune response**						
214450_at	0.011	2.52	n.c.	CTSW	cathepsin W	NM_001335
206082_at	0.007	1.60	n.c.	HCP5	HLA complex P5 (non-protein coding)	NR_040662
216542_x_at	0.007	1.50	n.c.	IGHA1	immunoglobulin heavy constant alpha 1	AJ275355
208071_s_at	0.011	1.50	n.c.	LAIR1	leukocyte-associated immunoglobulin-like receptor 1	NM_002287
217513_at	0.005	−1.65	n.c.	MILR1	mast cell immunoglobulin-like receptor 1	NM_001085423
208759_at	0.011	1.50	n.c.	NCSTN	nicastrin	AF240468
206804_at	0.011	1.91	n.c.	CD3G	CD3g molecule, gamma (CD3-TCR complex)	NM_000073
211373_s_at	0.012	−1.51	n.c.	PSEN2	presenilin 2	NM_000447
210479_s_at	0.008	1.79	n.c.	RORA	RAR-related orphan receptor A	NM_134261
209151_x_at	0.005	1.54	n.c.	TCF3	transcription factor 3	NM_003200
**Inflammatory response**						
217119_s_at	0.003	1.50	n.c.	CXCR3	chemokine (C-X-C motif) receptor 3	NM_001504
201348_at	0.005	1.63	n.c.	GPX3	glutathione peroxidase 3 (plasma)	NM_002084
207075_at	0.012	−1.80	n.c.	NLRP3	NLR family, pyrin domain containing 3	NM_004895
**Angiogenesis**						
216689_x_at	0.005	1.60	n.c.	ARHGAP1	Rho GTPase activating protein 1	NM_004308
208937_s_at	0.013	−1.87	n.c.	ID1	inhibitor of DNA binding 1	D13889
204626_s_at	0.006	1.62	n.c.	ITGB3	integrin, beta 3 (platelet glycoprotein IIIa, antigen CD61)	NM_000212
**MAPK cascade**						
210787_s_at	0.014	1.87	n.c.	CAMKK2	calcium/calmodulin-dependent protein kinase kinase 2, beta	AF140507
214786_at	0.003	1.77	n.c.	MAP3K1	mitogen-activated protein kinase kinase kinase 1	NM_005921
**Jak-stat signaling pathway**						
217199_s_at	0.015	1.54	n.c.	STAT2	signal transducer and activator of transcription 2, 113kDa	S81491
210995_s_at	0.011	1.55	n.c.	TRIM23	tripartite motif containing 23	AF230399
**Small GTPase signal transduction**						
216689_x_at	0.005	1.60	n.c.	ARHGAP1	Rho GTPase activating protein 1	NM_004308
218884_s_at	0.002	1.51	n.c.	GUF1	GUF1 GTPase homolog (S. cerevisiae)	NM_021927
209051_s_at	0.003	1.65	n.c.	RALGDS	ral guanine nucleotide dissociation stimulator	AF295773
**TCR signaling pathway**						
206804_at	0.011	1.91	n.c.	CD3G	CD3g molecule, gamma (CD3-TCR complex)	NM_000073
211373_s_at	0.012	−1.51	n.c.	PSEN2	presenilin 2	NM_000447
219724_s_at	0.012	2.07	n.c.	TESPA1	thymocyte expressed, positive selection associated 1	NM_014796
211902_x_at	0.010	2.06	n.c.	YME1L1	YME1-like 1 ATPase	NM_139312
**TGFB signaling pathway**						
221235_s_at	0.004	−1.50	n.c.	TGFBRAP1	transforming growth factor, beta receptor associated protein 1	NM_004257
205187_at	0.006	1.60	n.c.	SMAD5	SMAD family member 5	AF010601
214786_at	0.003	1.77	n.c.	MAP3K1	mitogen-activated protein kinase kinase kinase 1	NM_005921
203988_s_at	0.008	1.64	n.c.	FUT8	fucosyltransferase 8 (alpha (1,6) fucosyltransferase)	NM_004480
**Notch signaling pathway**						
203988_s_at	0.008	1.64	n.c.	FUT8	fucosyltransferase 8 (alpha (1,6) fucosyltransferase)	NM_004480
208759_at	0.011	1.50	n.c.	NCSTN	nicastrin	AF240468

**Table 7:** Biological processes that were significantly enriched in genes corrected by the treatment.

AS Patients-Group 1		AS Patients-Group 2	
Biological Process	*p* Value	Biological Process	*p* Value
activation of immune response	0.014	leukocyte activation	0.034
positive regulation of immune system process	0.002	lymphocyte activation	0.050
positive regulation of leukocyte activation	0.005	T-cell activation	0.038
positive regulation of lymphocyte activation	0.002	positive regulation of B-cell activation	0.015
positive regulation of lymphocyte proliferation	0.023	NLRP3 inflammasome complex assembly	0.006
lymphocyte activation involved in immune response	0.046	angiogenesis	0.002
T-cell activation	0.005	response to transforming growth factor beta	0.010
positive regulation of T-cell activation	0.005	G-protein coupled receptor signaling pathway	0.031
cellular response to unfolded protein	0.001		
positive regulation of B-cell activation	0.041		
B-cell cytokine production	0.024		
inflammatory response	0.026		
response to oxidative stress	0.022		
positive regulation of cytokine production	0.012		
positive regulation of type I interferon production	0.017		
toll-like receptor signaling pathway	0.039		
positive regulation of interleukin-2 production	0.001		
regulation of adaptive immune response based on somatic recombination of immune receptors	0.013		

## Supplementary Materials

The following are available online at www.mdpi.com/2073-4425/8/4/127/s1. Table S1: Clinical features of the patients enrolled in the study, Table S2: Genes modulated in AS patients before treatment versus healthy controls, Table S3: Enriched biological processes and pathways related to the eight pre-treatment modules, Table S4: Genes modulated in AS patients after treatment versus healthy controls, Table S5: Enriched biological processes and pathways related to the seven after -treatment modules, Table S6: Genes modulated in AS patients, group 1 before treatment versus healthy controls, Table S7: Genes modulated in AS patients, group 2 before treatment versus healthy controls, Table S8: Genes modulated in AS patients, group 1 after treatment versus healthy controls, Table S9: Genes modulated in AS patients, group 2 after treatment versus healthy controls.
