# Supplementary material for: Gene Expression Analysis before and after Treatment with Adalimumab in Patients with Ankylosing Spondylitis Identifies Molecular Pathways Associated with Response to Therapy
Source: Genes (Basel). 2017 Apr 24;8(4):127. doi: 10.3390/genes8040127 (PMC5406874; doi:10.3390/genes8040127)
Supplement: Supplementary file 1 [file genes-08-00127-s001.zip › Table S1 def.doc]

**Table S1. Clinical features of the patients enrolled in the study.**

| Patients used for the gene array study | 10 |
| --- | --- |
| Sex Male/female | 7/3 |
| Age at diagnosis (years) | 39,4±3,1 |
| Age at enrollment (Years) | 51,8±11,2 |
| Time from onset symptoms to diagnosis | 3,1±2,6 |
| Time from diagnosis to biological agents treatment beginning | 3,8±0,8 |
| Skeletal involvement  - Axial  - Peripheral | 10  4 |
| Association to HLA-B27 |  |
